# Equity in Transplantation Access in Nepal: An Analysis of Gender, Geographic, and Caste-Based Disparities in Transplants

**DOI:** 10.3389/ti.2023.11635

**Published:** 2023-11-30

**Authors:** Dibya Singh Shah, Midhan Shrestha, Bikash Khatri, Santosh Chhetri, Kalpana Shrestha, Sangita Sedhai, Upendra Joshi, Manish Gautam

**Affiliations:** ^1^ Department of Nephrology and Transplantation Medicine, Institute of Medicine, Tribhuvan University Teaching Hospital, Kathmandu, Nepal; ^2^ Bir Hospital, Kathmandu, Nepal; ^3^ KIST Medical College, Kathmandu, Nepal; ^4^ Shahid Dharmabhakta National Transplant Centre, Bhaktapur, Nepal; ^5^ Grande International Hospital, Kathmandu, Nepal; ^6^ Anweshan Pvt. Ltd., Kathmandu, Nepal

**Keywords:** equity, kidney transplant, Nepal, barriers, accessibility

## Abstract

Transplantation is a lifesaving modality for addressing various organ failures. While kidney transplant services became available in Nepal in 2008, the introduction of liver transplantation is more recent. The government provides financial assistance to support lifelong dialysis and kidney transplantation. The importance of equitable access to transplantation cannot be overemphasized. This study aims to examine the equity in accessing transplantation services. This retrospective observational study encompasses patients who underwent kidney transplantation up until December 2022 across five major hospitals. Through standardized data collection and analysis, we evaluated the distribution of recipients based on gender, caste/ethnicity, and geographic location. A total of 2040 kidney transplantations were performed during the period. Notably, 79% of the recipients were men and, interestingly, 70% of the donors were women. Geographically, the highest proportion (31.8%) of recipients were from Bagmati, while the lowest (l2.8%) were from Karnali. Regarding caste and ethnicity, Janajatis accounted for 31% and Chhetris for 22.9%; Madhesis were lowest at 8.12%. Only 17 liver transplantations were conducted during the same period. Although access to kidney transplantation exists in Nepal, this study highlights persistent disparities. Women, rural and remote populations, as well as specific ethnic and caste groups encounter barriers to accessing transplantation services.

## Graphical Abstract

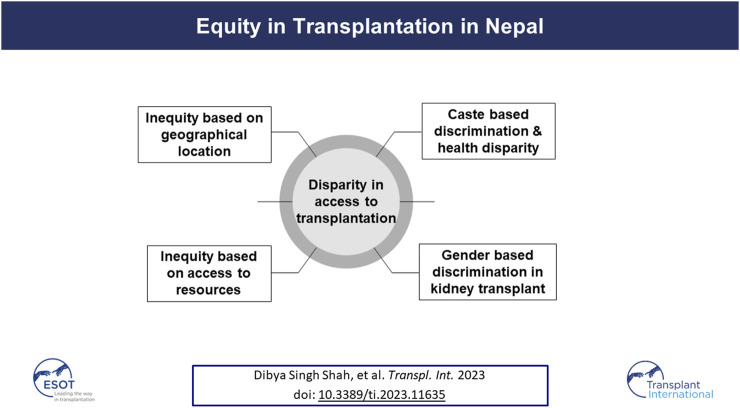

## Introduction

According to the World Health Organization (WHO), health equity represents the ideal state in which each individual possesses a fair opportunity to reach their optimal health potential. Transplantation serves as a life-saving treatment for numerous end-stage organ failures, enhancing both survival rates and quality of life while also proving to be economically viable. The importance of equitable access to this critical procedure cannot be overstated.

In a low-resource setting like Nepal, realizing this ideal state of equity comes with numerous challenges. Key obstacles include lack of infrastructure, limited human resources, low expenditure in healthcare, sociocultural behavior, and a substantial population living in remote rural areas.

Despite Nepal’s recent elevation to a lower middle-income country status in the World Bank’s latest country classification [1], a significant 79% of the population remain concentrated in rural areas.

The population of Nepal, as of 9 March 2021, was 29.16 million, divided across the country’s seven provinces, which cover 147,181 square kilometers. According to the national Census of 2021, Koshi accounts for 17.01% of the total population; Madhesh 20.97%; Bagmati 20.97%; Gandaki 8.46%; Lumbini 17.56%; Karnali 5.79%; and Sudurpaschim constitutes 9.24% of the country’s total population [2].

Nepal has identified 142 caste/ethnic groups. Among them, Brahmins comprise 11.29%, Chhetris 16.45%, Newars 4.60%, Janajatis (indigenous population) 36.04%, Dalits 12.38%, and Madhesis 19.24% of the country’s total population [3].

### Healthcare System

Healthcare in Nepal comprises a hybrid model encompassing both public and private sectors. The country’s healthcare system is predominantly reliant on out-of-pocket expenditure, with public hospitals offering services at relatively low costs. With a *per capita* GDP of USD 1,037, the World Bank’s 2022 data indicates that healthcare expenditure accounts for 4.45% of the 59 GDP [4].

In an effort to alleviate the burden of healthcare costs for citizens facing financial constraints, the Government of Nepal provides subsidies through the Disadvantaged Citizens Medical Treatment Fund. This initiative covers eight chronic conditions: cardiovascular diseases, cancer, renal failure, Alzheimer’s disease, Parkinson’s disease, head and spinal cord injury, sickle-cell anemia, and stroke [5].

The World Health Organization has identified universal health coverage as a key approach in reducing equity gaps within a country, with social health insurance as a recommended mechanism. Nepal’s legislative parliament endorsed the National Health Insurance bill on 10 October 2017 [6]. The governing body for the bill is the National Health Insurance Board, which aims to achieve universal health coverage by 2030.

### Non-Communicable Diseases

The burden of non-communicable diseases is on a steep ascent, driven by the increasing prevalence of diabetes and hypertension. This surge is attributed to shifts in lifestyle and dietary habits, as well as heightened exposure to chemicals and medications. While an official registry for end-stage organ failure is absent, based on the global scenario of end-stage kidney diseases, the estimated annual incidence stands at 100 cases per million population. Given the late stage presentation of diseases in the South Asian region, the actual prevalence could potentially surpass this estimate [7].

### Transplantation in Nepal

Although corneal transplantation started in Nepal in the 1980s, the history of solid organ transplantation is more recent. The first piece of legislation, the Human body transplantation Act, came in 1998. The country’s first successful solid organ transplantation was kidney transplantation, which was performed in August 2008 at the Institute of Medicine, Tribhuvan University Teaching Hospital [8]. The transplantation program is mainly centered on living donor transplants. The eligibility criteria for organ donors are strictly defined by law, limiting donation to close relatives, and the present law strictly prohibits unrelated organ donations [9]. The first amendment of the 1998 Act was made in 2016 with the inclusion of brain death criteria, Pair exchange and some extension of the related donation. The first brain death kidney transplantation was carried out in the same year at Sahid Dharma Bhakta Organ Transplant center. The first liver transplantation was started in 2017 with the assistance of a Korean liver transplant team, and there are currently three centers performing liver transplantation, but the program is dependent upon the visiting expert team from India.

The government provides around USD 5,000 per patient to cover kidney transplantation expenses and 1 year’s worth of immunosuppressive medication in government hospitals, facilitated through the Disadvantaged Citizens Medical Treatment Fund. Additionally, a provision of up to USD 900 per year for post-transplantation medications is extended to those under the coverage of the national health insurance policy. However, despite this support, a substantial number of patients still face barriers in accessing transplantation. Consequently, this study seeks to examine the current status of kidney transplantation and equity in access to this life-saving treatment modality within Nepal.

## Materials and Methods

This study follows a retrospective observational design. It was reviewed and approved by the Institutional Review Committee of the Institute of Medicine [R. no. 551 (6-11) E2].

The study’s scope included all recipients of kidney transplantation until December 2022 across five major hospitals. The list of hospitals authorized for transplantation, and the total number of transplantations conducted at these centers until December 2022, was obtained from the Department of Health Services. Standardized data encompassing gender, ethnicity, caste, place of residence, and donor relation was collected from hospital records through relevant departments.

The collected data was analyzed to assess the distribution of gender, geographical location, caste, and ethnicity in relation to transplantation access.

Additionally, data regarding the number of dialysis centers, hemodialysis machines, registered nephrologists, and transplant surgeons were obtained from records maintained by the Department of Health Services, respective dialysis units, and the Nepal Society of Nephrology.

Subsequent analysis of this data aimed to reveal the distribution of facilities and access to transplantation based on gender, ethnic groups, caste, and geographical locations.

## Results

Until December 2022, a total of 12 centers had obtained permission for kidney transplantation, with four centers approved for liver transplantation. Apart from two centers in Koshi province and one in Lumbini for kidney transplants, along with one center in Lumbini for liver transplant, all the other centers were located in Bagmati.

Among these centers, only five were actively conducting kidney transplantations, while the rest ceased operations after initial procedures. Regarding liver transplantation, a total of 17 liver transplantations have been performed. Among them, five were carried out in Lumbini province and the rest in Bagmati province. Only two transplantations were from deceased donors. These cases were performed in Nepal with assistance of transplant teams from Korea and India. With the exception of two recipients, all other recipients were men. Among all the donors, only two donors were men. The common donors were sisters, wives, and daughters.

**FIGURE 1:**
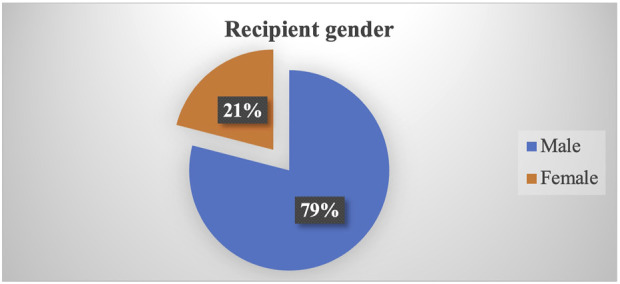
Proportion of recipient gender.

**FIGURE 2:**
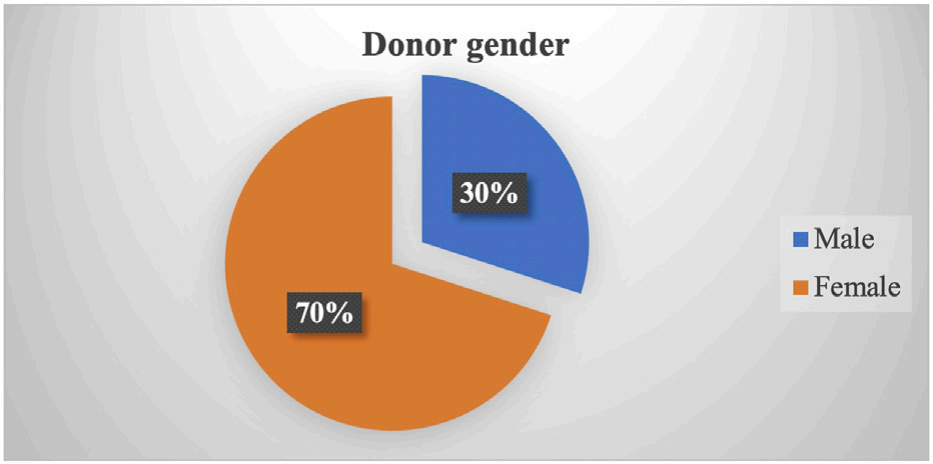
Proportion of donor gender.

From August 2008 to December 2022, a total of 2,040 kidney transplantations were carried out. Of these, 2022 (99.11%) occurred in five hospitals (three public and two private) located in Bagmati. Among recipients, 79% were men whereas women constituted 70% of donors (Figures 1, 2). The predominant transplantation type was living donor (barring eight cases), with mothers and wives being the most common donors.

**FIGURE 3:**
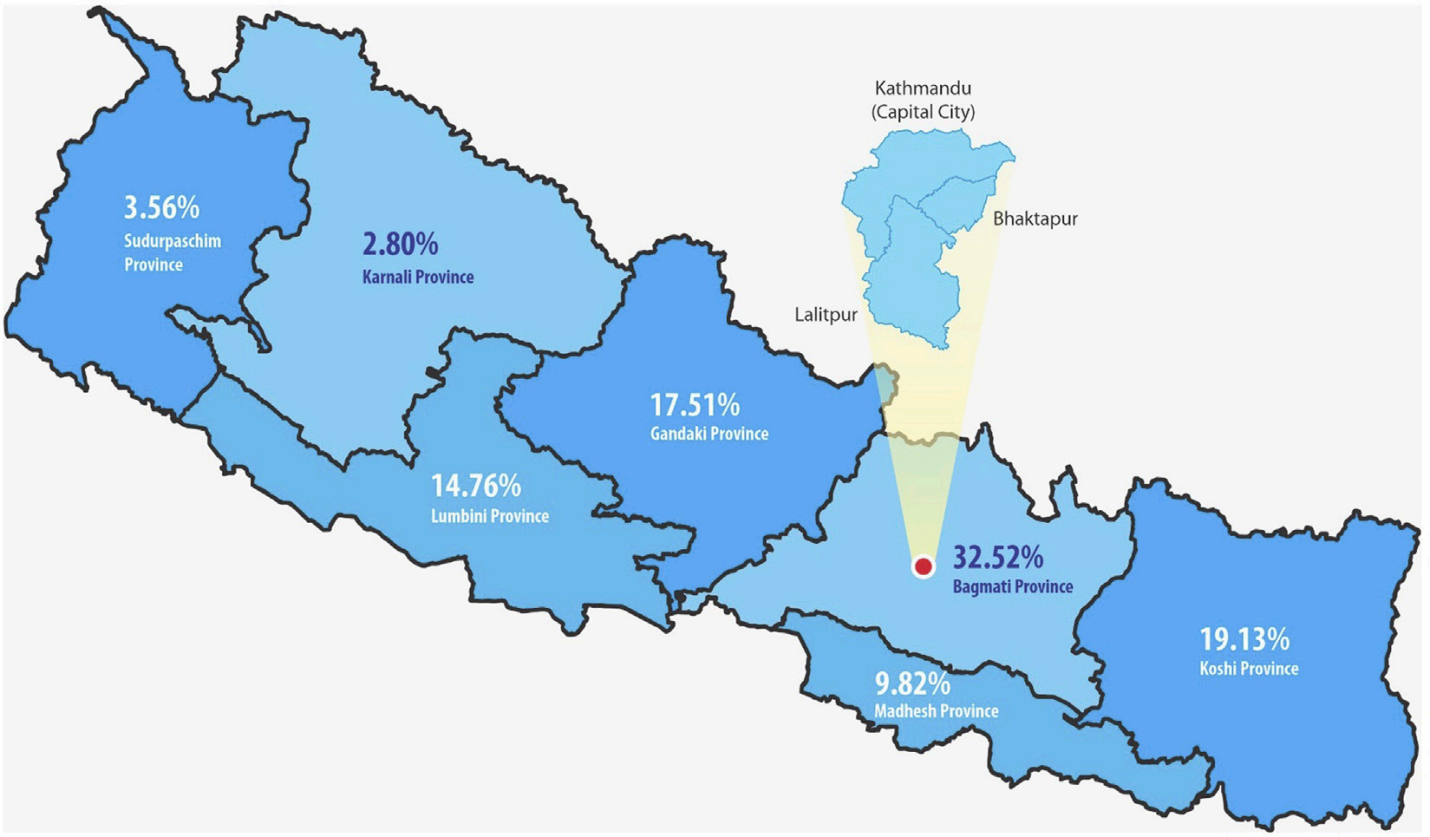
Transplant recipients in different provinces of Nepal.

**FIGURE 4:**
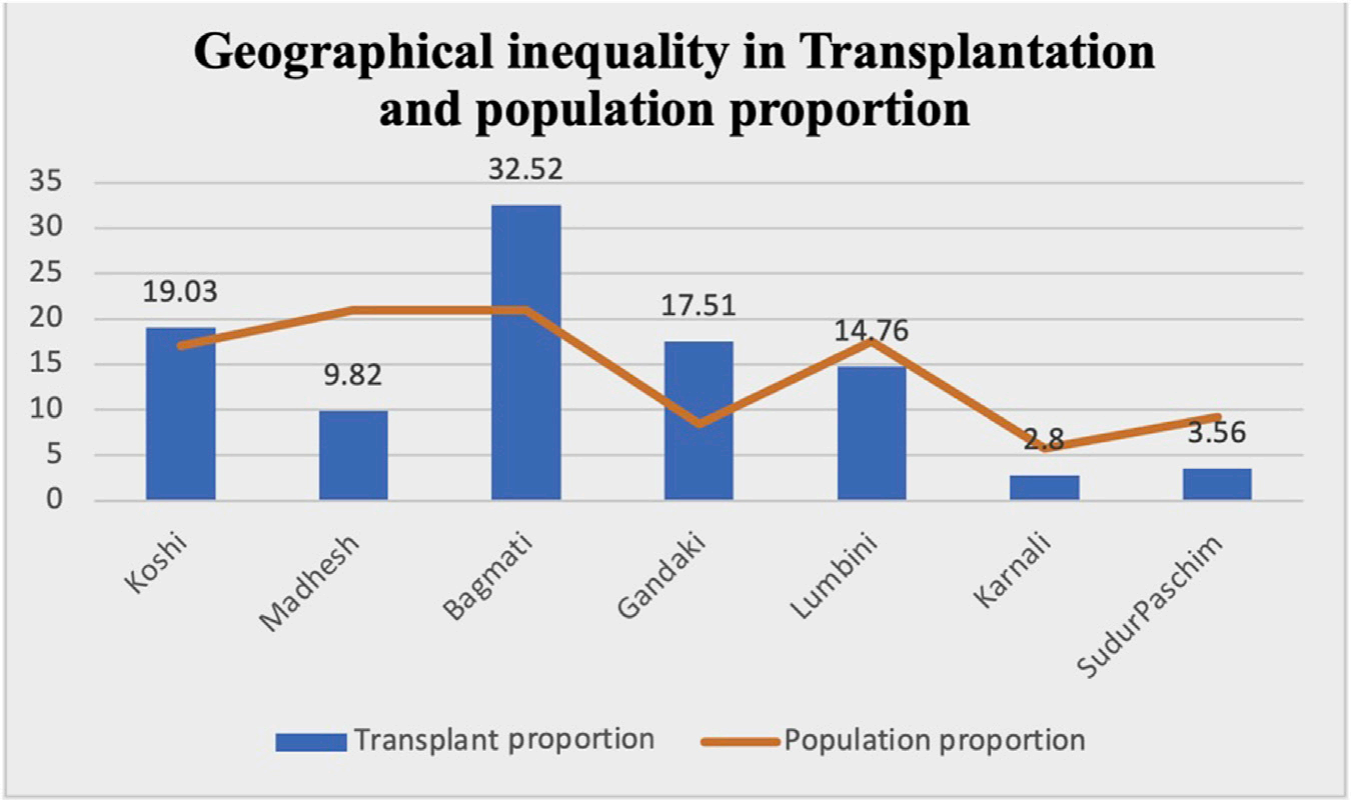
Proportion of transplant patients as compared to population proportion in different provinces.

Distribution by region showed that the highest proportion (32.52%) of kidney transplant recipients were from Bagmati, which consists of only 20.97% of the population. Subsequently, 19.3% were from Koshi (17.07% of the population), 17.51% from Gandaki (8.46% of the population), 14.76% from Lumbini (17.56% of the population), 9.82% from Madhesh (20.97% of the population), 3.56% from Sudurpaschim (9.24% of the population), and the least 2.80% from Karnali (5.79% of the population) (Figures 3, 4).

**FIGURE 5:**
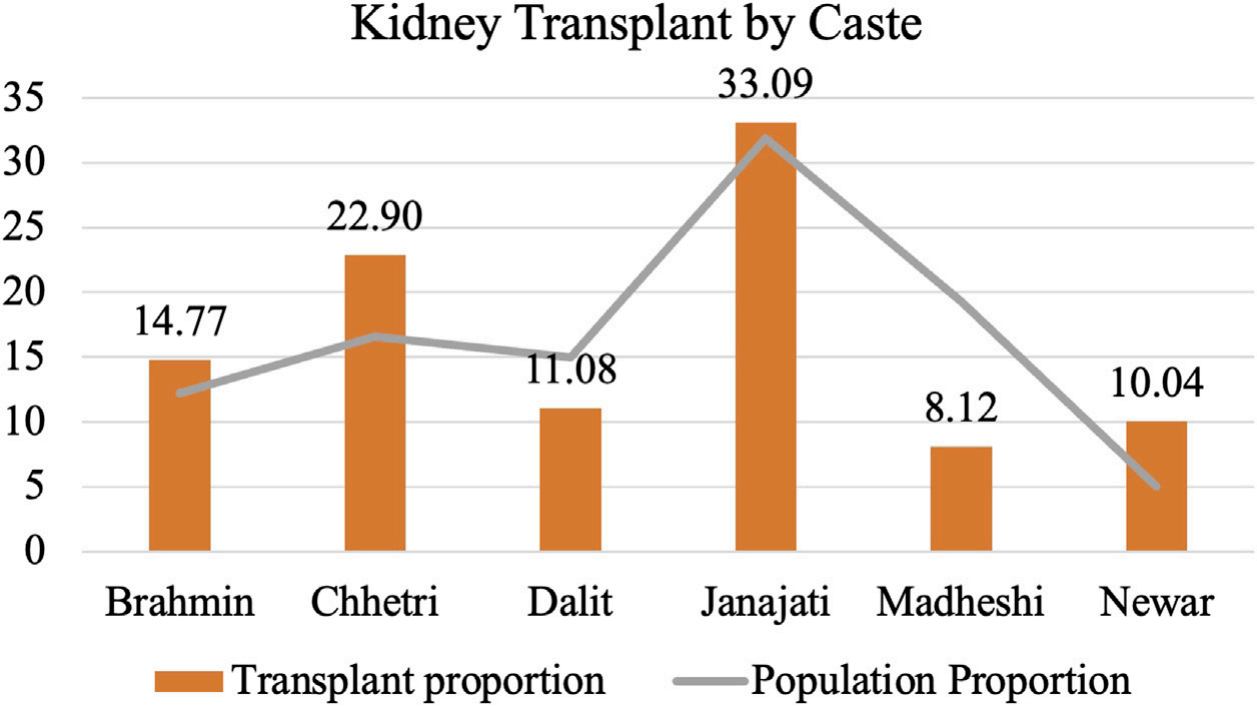
Proportion of transplant recipients by caste as compared to population proportion.

Regarding caste and ethnicity, the recipients comprised 31% Janajatis, 22.90% Chhetris, 14.77% Brahmins, 10.04% Newars (locals of the Kathmandu Valley), 11.08% Dalits, and the lowest proportion (8.12%) were Madhesi (people from the Terai), despite consisting 19.3% of the total population and being situated in a geographically accessible region (Figure 5).

According to the Nepal Society of Nephrology, the country has 69 registered nephrologists, with 52 practicing within Kathmandu Valley located in Bagmati Province and 17 outside. The total number of licensed kidney transplant surgeons stood at 12, of which 11 were active within Kathmandu Valley in Bagmati Province.

**FIGURE 6:**
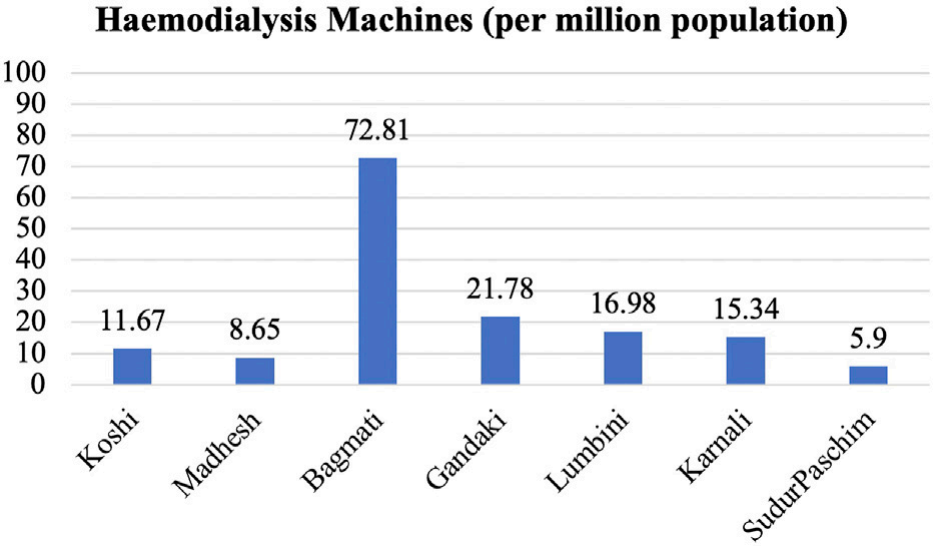
Distribution of HD machines according to provinces.

A regional comparison of hemodialysis machines per million population showed the highest concentration in Bagmati, at 72.81 machines, followed by 21.78 per million in Gandaki, 16.98 per million in Lumbini, 15.34 per million in Karnali, 11.67 per million in Koshi, 8.65 per million in Madhesh, and the lowest, 5.9 per million, in Sudurpaschim (Figure 6).

**FIGURE 7:**
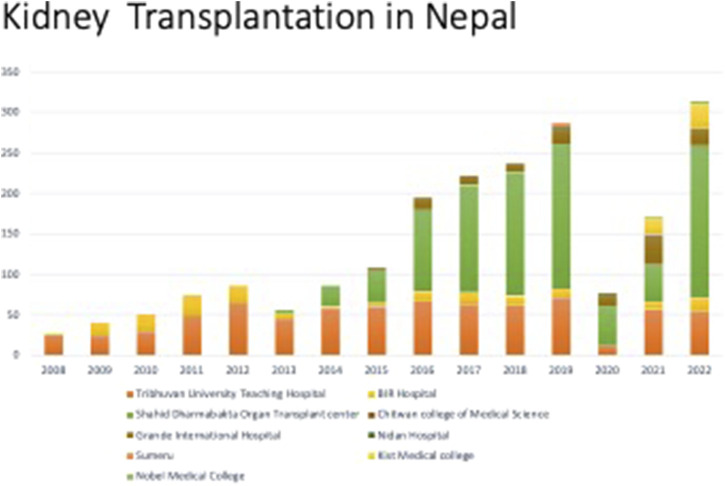
Renal transplantations performed at different centers in Nepal.

**FIGURE 8:**
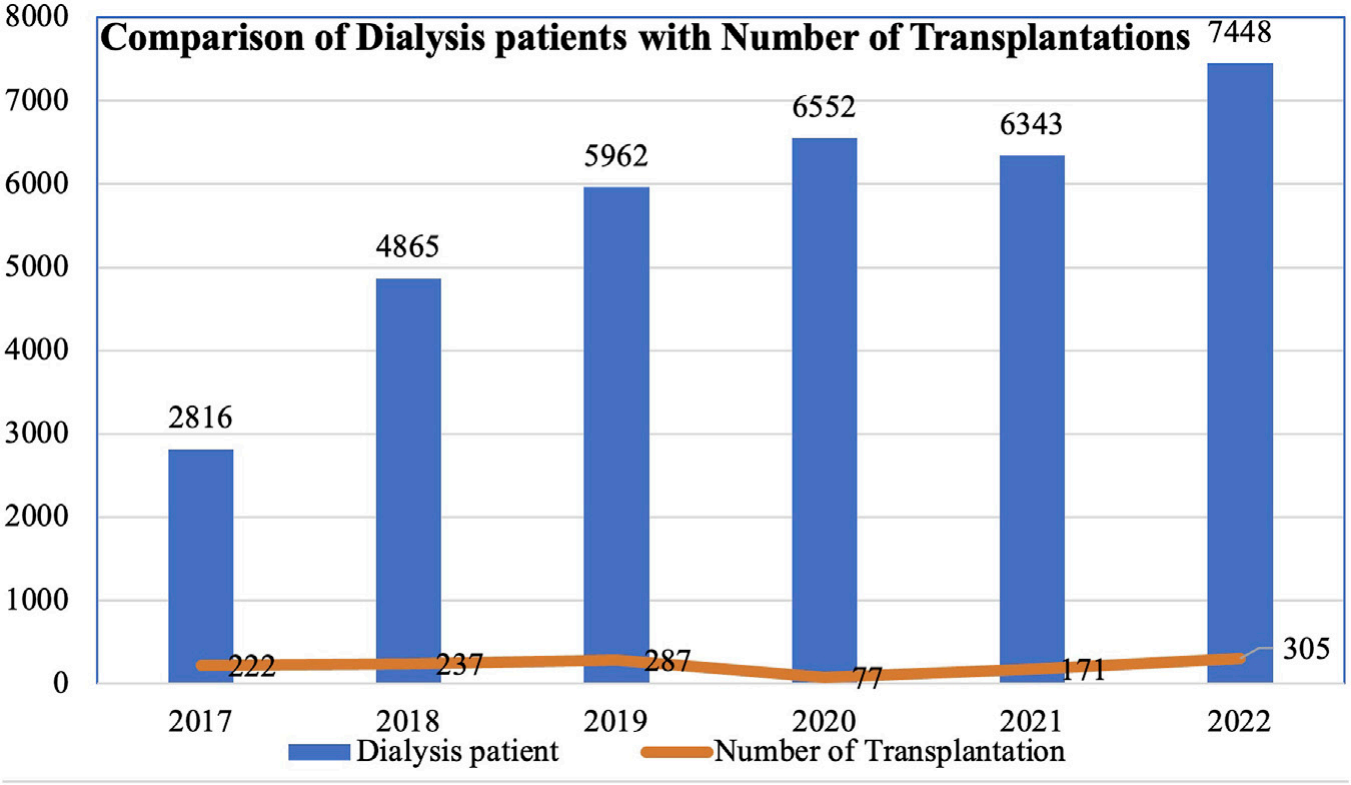
Number patients on dialysis and renal transplantations performed per year.

Since 2008, there has been a steady rise in the number of annual kidney transplantations, barring the years 2020 and 2021 when the COVID-19 pandemic led to a decline. Over 300 kidney transplantations were performed in 2022 alone (Figure 7). However, this figure remains insufficient when contrasted with the number of individuals undergoing dialysis (Figure 8).

## Discussion

### Inequity Based on Geographical Location

In our study, the majority of recipients (32.52%) were from Bagmati, which encompasses 20.97% of the total population of the country. However, despite Madhesh having an equal proportion of the population as well as being geographically convenient, only 9.82% of the recipients hailed from the province. Conversely, Gandaki, comprising a mere 8.46% of the population, yielded a higher recipient percentage of 14.76% of recipients. The lowest rate of recipients, at 2.8%, was in Karnali.

This disparity stems largely from the clustering of specialists, dialysis centers, and machines within urban centers. Rural residents often must either migrate toward cities or undertake extensive journeys to access maintenance hemodialysis. While continuous ambulatory peritoneal dialysis could be a potential solution, difficulties in accessing peritoneal dialysis fluid and high transportation costs to remote areas render this modality less feasible for patients.

Another contributing factor to this disparity lies in the limited number of public hospitals performing regular transplantations, with waiting time stretching between three to 6 months. Consequently, individuals often need to temporarily relocate to the capital city, Kathmandu, for work-up, surgery, and follow-up care. This adds to the cost, including the loss of wages for accompanying family members. Geographical disparities in transplantation accessibility have also been reported in studies from other parts of the world [10, 11].

### Inequity Based on Access to Resources

Although the number of transplantations has increased steadily over the years, with around 250 to 300 procedures carried out annually, this figure remains disproportionately low when compared to the demand for transplantation in the country. Many are compelled to endure lifelong dialysis due to scarcity of living donors. In Nepal, the organ procurement law restricts donations to close relatives. Unfortunately, many patients discontinue dialysis due to various socioeconomic reasons. Some patients even travel abroad to seek unrelated transplantation. With regards to liver transplantation, a limited number of procedures have been conducted with the assistance of foreign experts. For those who possess the financial means, traveling abroad for liver and other organ transplants is an option, albeit one that remains inaccessible to the majority of the population.

Notably, although the Amendment of the Human body organ Transplantation Act with the inclusion of brain death criteria was approved in 2016, only four donations have happened so far. The deceased donor program has not developed as a national program, as it is centered in only one government hospital, in which patients voluntarily register their names for the deceased organs.

However, a digitalized format for a central wait-listing platform has recently been developed by the department of health including all the transplant centers in Nepal. More importantly, there is an intense need for dedicated National Organ transplantation office under the government for the promotion, coordination, and implementation of the deceased organ transplantation program in Nepal, which will not only reduce the gap of the present demand but also open the door to move forward with other organ transplantations.

### Gender-Based Discrimination in Kidney Transplantation

A notable gender disparity is evident in our study, with 79% of recipients being men and 70% of donors being women. Mothers and wives emerge as the most common related donors. The data shows that women are less likely to be referred for kidney transplantation and subsequently face greater challenges in securing donors, resulting in lower likelihood of undergoing transplantation [12]. This discrepancy can be attributed to the prevailing patriarchal societal norms, by which men are commonly seen as the primary earners and women the homemakers. As a result, women are often obliged to donate organs for the greater benefit of the family [13].

Furthermore, as men are seen as protectors and assets to the family, family members discourage them from donating organs. This discrimination is not unique to Nepal and other South Asian countries; some degree of discrimination exists even in developed nations [14, 15].

### Caste-Based Discrimination and Health Disparity

Within our study, Madhesi and Dalit communities exhibit low representation in accessing kidney transplantation, whereas Brahmins and Chhetris have higher representation. Janajati communities also hold a relatively greater representation compared to their respective population proportions. We generalize this as a part of general health discrimination among different groups. A confluence of factors, especially racial discrimination in areas such as housing, education, nutrition, healthcare, and employment, contribute to this discrepancy. Similar to other minorities, Dalits in Nepal tend to have lower incomes, less education, and live in areas with limited access to nutritious food. This further translates to restricted access to diagnosis and treatment for chronic health issues.

Inequalities in various aspects of End Stage Kidney Disease have been well-documented, even in developed countries like the United Kingdom. Ethnic minorities tend to experience a more rapid progression from Chronic Kidney Disease (CKD) to End Stage Kidney Disease. Moreover, minority groups face challenges in accessing timely care and frequently experience late referrals to specialist renal care. The difference between ethnic groups occurs at multiple points and across diverse outcomes throughout the kidney care system. The combination of individual factors and system-related variables affects ethnic groups differently, indicating a need for culturally intelligent policies informed by research to address the needs of disadvantaged populations [16].

Interestingly, a study conducted by Poudyal et al. in Nepal identified the so-called Dalit caste as an independent risk factor for CKD. The study’s overall CKD prevalence was 6.0%, and factors independently associated with CKD included older age, Dalit caste, hypertension, diabetes mellitus, elevated total cholesterol levels, and an increased waist-to-hip ratio [17].

Global studies highlight that ethnic minorities with End Stage Kidney Disease are disproportionately represented in transplantation modalities. This stems from various factors linked to the transplantation systems of different countries. These factors include listing rates for transplantation, movement from the waiting list to transplantation, variations across transplant centers, pre-dialysis care differences, and cadaveric and live donation rates, all of which indicate disparities when comparing ethnic minorities with majority populations [18].

## Conclusion

Nepal’s only established organ transplantation program is kidney transplantation. Despite notable progress in this field, significant disparities in access persist, with resources concentrated in urban centers. This leaves rural, vulnerable, and marginalized groups underserved.

The solution lies in restructuring healthcare for regional autonomy, implementing uniform universal healthcare, and promoting deceased donor programs, which can help bridge gaps and address disparities.

## Data Availability

The original contributions presented in the study are included in the article/supplementary material, further inquiries can be directed to the corresponding authors.
